# From channelrhodopsins to optogenetics

**DOI:** 10.1002/emmm.201202387

**Published:** 2013-01-22

**Authors:** Peter Hegemann, Georg Nagel

**Affiliations:** Institute of Biology, Experimental Biophysics, Humboldt-University of BerlinBerlin, Germany; Department of Botany I, Julius-von-Sachs-Institute for Biosciences, University of WürzburgWürzburg, Germany

We did not expect that research on the molecular mechanism of algal phototaxis or archaeal light-driven ion transport might interest readers of a medical journal when we conceived and performed our experiments a decade ago. On the other hand, it did not escape our attention that channelrhodopsin is helping an ever-increasing number of researchers to address their specific questions. For example, the channelrhodopsin approach is used to study the molecular events during the induction of synaptic plasticity or to map long-range connections from one side of the brain to the other, and to map the spatial location of inputs on the dendritic tree of individual neurons. The current applications have been summarized in a number of recent reviews (Fenno et al, 2011; Yizhar et al, 2011; Zhang et al, 2011). Here, we give personal insight into the history of the discovery of channelrhodopsin and a biophysical perspective on this remarkable class of proteins that has been the main topic of our research since the 1990s.

## Channelrhodopsin's roots

The discovery of channelrhodopsin is based on two quite different research fields, studies on living algae and experiments on reconstituted microbial rhodopsins.

**Figure 1:**
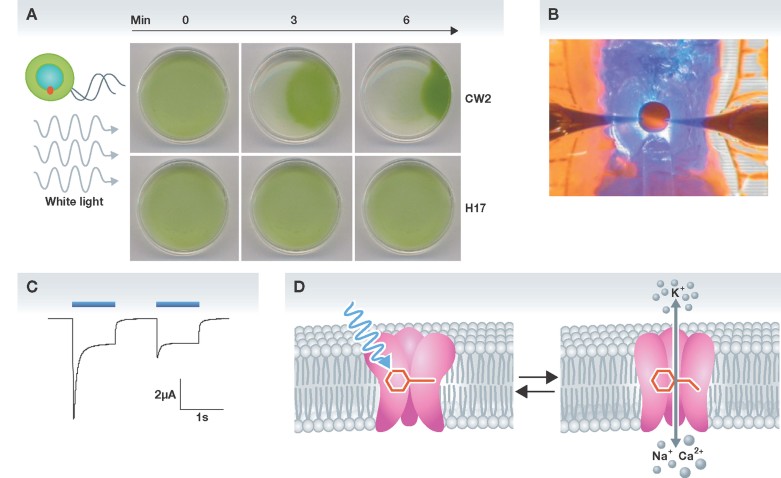
The discovery of channelrhodopsins Phototaxis of the *Chlamydomonas* wild type strain CW2 and the channelrhodopsin-defective mutant H17.Electrophysiological recording from oocytes (in the centre, with two electrodes, left and right) allows investigation of light-induced (via light guide, below oocyte) currents, *e.g.* mediated by rhodopsins.Light-induced currents mediated by channelrhodopsin-2 (two illuminations for 1 s, blue bars) at −100 mV.Model of channelrhodopsin opening, following light absorption and isomerization of covalently bound retinal.

A number of researchers have characterized the swimming behaviour and light responses of motile microalgae over at least 140 years (Fig 1A). Early studies on green microalgae root back to L.G. Treviranus (Treviranus, 1817) and behavioural responses were described by A. Famintzin from St. Petersburg University in 1878 (Famintzin, 1878). During helical swimming of the green alga *Chlamydomonas*, its orange eye signals to the flagella to alter the flagellar beating plane (Mast, 1916). Researchers at Stanford University implicated Mg^2+^ and Ca^2+^ in the behavioural responses and identified the role of Ca^2+^ influx in flagellar beat frequency changes (Halldal, 1957, Schmidt & Eckert, 1976). Then Oleg Sineshchekov from Moscow State University recorded electrical light responses from *Haematococcus pluvialis*, an alga known for the production of the antioxidant Astaxanthine (Litvin et al, 1978). Oleg used a suction pipette technique applied at the time by Dennis Baylor for recording photocurrents from bovine photoreceptor rods and cones. But Oleg's publication gave no hints about the type of photoreceptor involved. Kenneth W. Foster however, a physicist at Mount Sinai School of Medicine re-analysed published action spectra for phototactic movement of algae and postulated that the sensory photoreceptor is rhodopsin (Foster & Smyth, 1980). Ken substantiated his claim by restoring behavioural light responses in blind algae by complementation with retinal and retinal analogues (Foster et al, 1984). However, the photoreceptor field did not really understand the importance of the claim and progress remained slow. Years later, Peter Hegemann's former graduate student Hartmann Harz recorded photocurrents from *Chlamydomonas* by revitalizing Oleg's suction pipette technique for a *Chlamydomonas* cell wall-deficient mutant. He recorded action spectra, which led to the proposal that the photocurrents were mediated by a rhodopsin, the photoreceptor that also mediates phototaxis and phobic responses (Harz & Hegemann, 1991). The ultra-fast appearance of the photoreceptor current suggested that the photoreceptor and ion channel were intimately linked, forming a single protein complex (Braun & Hegemann, 1999).

»…the photoreceptor and ion channel were intimately linked, forming a single protein…«

In parallel, biophysicists had characterized the precise nature of light-regulated ion transport across cellular membranes. Some of these studies started with investigations on animal rhodopsin and even suggested rhodopsin-mediated light-induced calcium entry with rhodopsin itself as the carrier for calcium (Cone, 1972). Several decades later, we know that animal-type rhodopsins are G protein-coupled receptors indirectly modulating ion channel activity via signalling molecules. A big surprise was the discovery of the first rhodopsin in a procaryote (Oesterhelt & Stoeckenius, 1971). This ‘bacteriorhodopsin’ is a light-driven proton pump and meanwhile the best-studied membrane protein. For our considerations important are the electrical studies on bacteriorhodopsin (BR) which started in the mid-1970s (Bamberg, 1977; Herrmann & Rayfield, 1976), confirming and detailing the light-activated proton pumping function of BR. One of the big advantages of bacteriorhodopsin was its ready availability and its unusual stability at room temperature. Paradigms changed with the tremendous success of gene technology, when proteins could be investigated without the need of protein purification, simply by expressing the protein in the cellular system of choice. Even though a lot was already known about BR, the exact voltage dependence of proton pumping was unclear. Therefore, Ernst Bamberg and Georg Nagel decided to study BR in the membrane of an animal cell, the oocyte of *Xenopus laevis* (Fig 1B). This gene transfer allowed the exact determination of the voltage dependence of light-activated proton pumping in a wide voltage range (Nagel et al, 1995). Later, the Cl^−^-pump halorhodopsin and the phototaxis-mediating sensory rhodopsins were also studied successfully in oocytes (Schmies et al, 2001).

## Discovery of channelrhodopsins

After many years of hard work, the Hegemann group had not succeeded in purifying the photoreceptors biochemically, due to the scarceness, instability, and heterogeneity of the proteins. A new approach needed to be initiated. In 2001, Suneel Kateriya in the Hegemann group identified novel DNA sequences that encoded for large microbial-type rhodopsins in a cDNA data bank from *Chlamydomonas*. To explore their function, our fruitful collaboration started when Georg Nagel expressed the two rhodopsins in *Xenopus* oocytes. We demonstrated that both DNAs encode directly light-gated cation channels. We named these new genes channelrhodopsin-1 (ChR1) and channelrhodopsin-2 (ChR2; Nagel et al, 2002, 2003; Fig 1C and D). These experiments were the undisputable proof for a completely new class of rhodopsins. During this collaboration we also expressed ChR2 in human kidney and other mammalian cells, showed large light-induced membrane depolarization, and suggested that ChR2 could also be used in other cells to depolarize these cells with light (Nagel et al, 2003).

## Transfer to neuroscience, take-off of optogenetics

Taking our suggestion into account, several groups began to work with ChRs, primarily with a truncated version of ChR2 that we had shown to be sufficient for light-gated cation conductance. The seminal publications appeared in 2005 and 2006 and came from the laboratories of Zhuo Pan, Karl Deisseroth, Stefan Herlitze, Hiromu Yawo and Alexander Gottschalk, who demonstrated the functionality of ChR2 in the retina of blind mice, hippocampal neurons, spine of living chicken embryos, PC12 cells, mouse brain slices and transgenic worms (Bi et al, 2006; Boyden et al, 2005; Ishizuka et al, 2006; Li et al, 2005; Nagel et al, 2005). These publications were the beginning of the field that we now term optogenetics. In this emerging field, researchers express light-activated proteins in well-defined cell subpopulations of a neuronal context and activate these cells by using short light pulses. Optogenetic studies started earlier, for example in Gero Miesenböck's and Rich Kramer's laboratories, when researchers implemented photosensitive actuators into host cells (Banghart et al, 2004; Zemelman et al, 2002). These systems turned out to be either too complicated or the electrical response was too slow. However, neuroscientists were sensitized for approaches with light-modulated proteins. The recent success of optogenetics is to a large part based on the simplicity of the merely 315 amino acid long ChR2 fragment, which only needs easily available and cheap retinal as a co-factor. As the mammalian brain already contains retinal, no exogenous addition is required.

The success of ChR2 encouraged us and a number of neurobiologists to test halorhodopsin, a light-driven chloride importer and membrane hyperpolarizer, as an additional optogenetic tool for action potential suppression, which worked astonishingly well (Zhang et al, 2007).

»…demonstrated the functionality of ChR2 in the retina of blind mice, hippocampal neurons, spine of living chicken embryos, PC12 cells, mouse brain slices and transgenic worms…«

## The architectural design and function of channelrhodopsins

**Figure 2:**
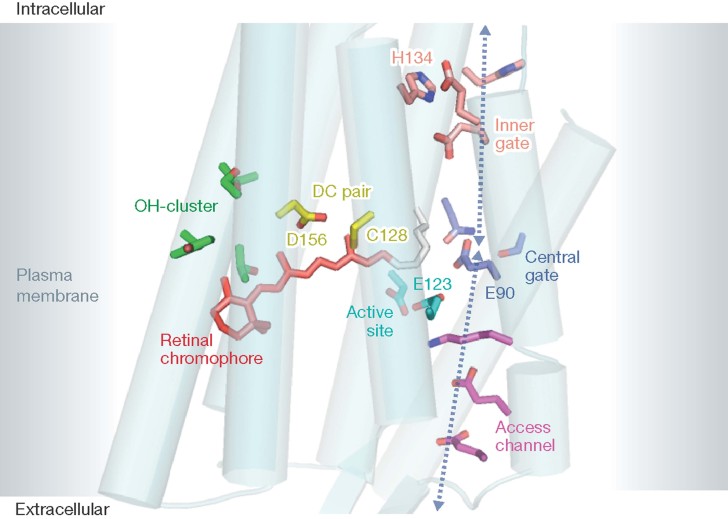
Cartoon of the Channelrhodopsin 7TM-fragment The structure is drawn according to the data of Kato et al (2012) with key residues shown in color: voltage sensor E123 (cyan), residues of the access channel (magenta), central gate (blue), and inner gate (orange), OH-cluster green, and the retinal Schiff base is seen in red.

ChRs are composed of seven trans-membrane helices that form the ion channel and a long C-terminal extension of unknown function, which is routinely omitted for optogenetic purposes. The light-absorbing chromophore retinal, a vitamin A derivative, is embedded within the hydrophobic center of the seven helices (Fig 2). The retinal is connected to a conserved lysine via a Schiff base linkage (C=N), which is protonated to shift the absorption into the visible range of the spectrum. The colour of the protonated retinal Schiff base (RSBH^+^) is fine-tuned by the distance of the negatively charged counter ion that together form the *active site* (Fig 2) and the location of a few polar residues around the retinal polyene chain. Light absorption by retinal leads to isomerization, followed by a protein conformational change and opening of the ion pore (Fig 1D). In the light-activated ion pumps, bacteriorhodopsin and halorhodopsin undergo similar conformational changes, which lead to active proton export and Cl^−^ import, respectively. Interestingly, internal and external pHs strongly influence ChR2 channel closing and recovery from desensitization (Nagel et al, 2003).

The structural changes are reversed during closure of the conducting pore and reversion to the dark state. We now know that this reaction path differs from the opening path and that the kinetics of dark state recovery is many orders of magnitudes slower. Besides the OH-cluster, two residues, C128 and D156 (DC-pair in Fig 2) are of fundamental importance for both channel opening and closing, and mutation of either residue results in a dramatic increase of the open state(s)' lifetime.

## Perspectives

Our expectations for future applications of ChR are high. However, ChRs show clear limitations, such as the small conductance. We may be able to widen the pore by molecular engineering, but presumably at the cost of destabilization and thermal activation in darkness. Selectivity can be changed towards higher or exclusive H^+^ conductance as found naturally in ChR from the halotolerant alga *Dunaliella salina* (Zhang et al, 2011). Likewise, ChR is tunable towards higher selectivity for monovalent or divalent cations. But greater selectivity for K^+^ over Na^+^, to be used for light-controlled hyperpolarization of host cells, will be very difficult to achieve. Moreover, the highly appreciated red-shifted absorption is limited to around 630 nm due to thermal activation (dark noise) of red light-absorbing rhodopsins even when synthetic retinal analogues are used as chromophores.

Despite these limitations, engineering of ChR and other microbial rhodopsins will progress and, moreover, countless ChR variants will be discovered from the hundreds of new algal genomes sequenced. Better solutions for targeting ChRs into membrane subareas will be found, directing them into organelles, making them bimodal switchable, controlling expression more accurately, and guaranteeing better turnover and photostability for retinal prosthesis and vision in bright light. ChRs will be further optimized for two-photon microscopy and many novel unprecedented variants will be identified. Moreover, ChRs may become commonly used analytical tools or even therapeutics for treating specific diseases.
